# Polymicrobial Periprosthetic Infection with *Pasteurella canis* Following Total Knee Arthroplasty: A Case Report

**DOI:** 10.3390/reports9040331

**Published:** 2026-09-29

**Authors:** Elena Cristina Gîrbovan, Alexandra Ioana Asztalos, Adrian Vlad Pop, Anca Meda Văsieșiu, Mircea Gîrbovan, Anca Cighir

**Affiliations:** 1 Department of Infectious Diseases, George Emil Palade University of Medicine, Pharmacy, Science and Technology of Târgu Mureș, 38 Gheorghe Marinescu Street, 540139 Targu Mures, Romania; 2 1st Infectious Diseases Clinic, Mures County Clinical Hospital, 89 Gheorghe Doja Street, 540233 Targu Mures, Romania; 3 Mureș County Clinical Hospital, 540233 Targu Mures, Romania; 4 Department of Microbiology, George Emil Palade University of Medicine, Pharmacy, Science and Technology of Târgu Mureș, 38 Gheorghe Marinescu, 540139 Targu Mures, Romania

**Keywords:** *Pasteurella canis*, periprosthetic joint infection, case report, zoonotic disease

## Abstract

**Background and clinical significance:***Pasteurella* spp. are Gram-negative coccobacilli, aerobic facultatively anaerobic, belonging to the *Pasteurellaceae* family. In humans, they are known to cause zoonotic diseases, which usually develop after dog or cat bites, licks or scratches. **Case presentation:** We present the case of a 61-year-old patient, with cardiovascular comorbidities, who presented to the Orthopedics Department, 38 days after knee replacement surgery, with recurrence of pain on walking, an antalgic gait with auxiliary support, and local signs of inflammation at the level of the left knee joint. The patient admitted to owning a dog who accidentally licked the post-surgical wound. The symptoms were suggestive of a post-surgical wound infection with extension to the periprosthetic area. A purulent discharge was collected from the wound during surgical debridement and sent to the microbiological laboratory for diagnosis, where *Pasteurella canis* was identified using MALDI-TOF MS. Antibiotic susceptibility testing (AST) was performed according to the EUCAST guidelines and empiric antibiotic treatment was started with Cefuroxime. During his future admissions to the Orthopedics and Infectious Diseases departments, two more samples were collected, in which *Pasteurella canis* was isolated, along with *Klebsiella oxytoca* (second sample), and *Klebsiella oxytoca* and *Pseudomonas aeruginosa* (third sample). Patient evolution was favorable due to the debridement of the wound and combined antibiotic treatment, with Ceftazidime and Gentamicin. **Conclusions:** Infections with *Pasteurella canis* are rare but slowly increasing due to more and more humans owning pets. The present case underlines the importance of interdisciplinary collaborations when rare pathogens are identified.

## 1. Introduction

*Pasteurella* spp. are Gram-negative coccobacilli, aerobic facultatively anaerobic, nonmotile, belonging to the *Pasteurellaceae* family. The genus has a worldwide distribution and can be found in the normal flora of the oral cavity and gastrointestinal tract of many animals [1,2].

In humans, most infections with *Pasteurella* spp. are attributed to cat or dog bites, licks or scratches. In immunosuppressed people and those diagnosed with chronic diseases, lesions can rarely develop after exposure to animals, without having a history of bites or scratches [3]. From the total number of animal bites, 3–18% of dog bites and 28–80% of cat bites become infected. Even more, 50% of dog bites and 75% of cat bites are associated with the presence of *Pasteurella multocida*, whereas *Pasteurella canis* is mainly associated with dogs and is the most common *Pasteurella* species recovered from infected dog-bite wounds [4]. Nevertheless, human infections caused by *Pasteurella canis* are reported far less often than those due to *P. multocida* [5].

Furthermore, the polymicrobial nature of *Pasteurella* spp. infection was also consistently reported, with co-isolated strains including *Staphylococcus aureus*, *Actinomyces* spp., *Streptococcus sanguinins* and *Dermatobia hominis* [6].

Infections with *Pasteurella canis* cover a wide variety of target organs, the most well-known being wounds [7] and soft tissue infections [8]. Other infections, such as bacteriemia [8,9], eye infections [3], respiratory infections [2], gastrointestinal infections [10], septic arthritis [11], osteomyelitis [12], peritonitis [13,14] and breast implant infections [15] are rarely described in the literature, and are limited to immunocompromised individuals.

Underlying conditions such as diabetes mellitus and cirrhosis and old age were commonly reported risk factors for bacteriemia, with a reported prevalence of 12.4% and a higher mortality rate (8.4%) [6].

Due to the routine prompt prophylactic treatment of animal bite wounds with antibiotics, pasteurellosis is a relatively uncommon cause of mortality in humans, even though deaths due to this infection have increased in the recent years [16].

Prosthetic joint infections caused by *Pasteurella* spp. are uncommon, although an increasing number of cases have been reported; a recent comprehensive review identified 57 cases published between 1975 and 2024, 56 of which were due to *P. multocida* [17]. Infection most often involves knee prostheses and follows bites, scratches or licks from cats or dogs. To our knowledge, only two cases of periprosthetic infection due to *P. canis* have been described [17,18]. Even though those infections are rare, they can be especially severe, as they require surgical revisions and have an increased mortality and morbidity [17].

Infections with *Pasteurella* spp. can pose diagnostic challenges, especially in atypical localizations. As the number of people who own pets increases every year, so does the incidence of this disease. Therefore, rising awareness about this pathogen is extremely important.

We report a case of polymicrobial periprosthetic infection with *Pasteurella canis* in a post-operatory wound after total knee arthroplasty.

## 2. Case Report

### 2.1. Patient Background

We present the case of a 61-year-old male patient, retired, from a rural area, with multiple comorbidities: hypertensive cardiomyopathy, grade III hypertension, aortic sclerosis, tricuspid regurgitation, mitral annulus calcification, and grade I obesity.

### 2.2. First Admission (Orthopedics)

He was admitted to the Orthopedics Clinic of the Mureș County Clinical Hospital for pain on walking and an antalgic gait without auxiliary support, being diagnosed with primary left gonarthrosis, Ahlback V stage, and secondary genu varum.

Local examination of the left knee revealed normally colored skin, global deformity of the joint, intra-articular crepitus, hypotrophy of the thigh and calf musculature, tenderness on palpation of the joint line and on patellar mobilization, pain-limited active and passive range of motion in the affected joint, active flexion of 100°, an extension deficit of approximately 5°, and varus deviation.

Left knee radiograph (weight-bearing anteroposterior and lateral views) revealed a narrowing of the medial femorotibial joint space with widening of the lateral femorotibial space, subchondral osteosclerosis in the weight-bearing area, osteophytosis of the femur, tibia, and patella, and varus deviation.

Surgical intervention was performed, consisting of total arthroplasty of the left knee with a cemented total endoprosthesis, posterior-stabilized, Stryker Triathlon type. The postoperative course was favorable, with drain removal at 24 h, active flexion in bed of approximately 90 degrees, and an extension deficit of 5 degrees. At 48 h postoperatively, a hematoma was evacuated at the distal 1/3 of the surgical wound, for which a compression dressing was applied, and movement restriction was recommended.

### 2.3. Second Admission (Orthopedics—Wound Complication)

The patient was discharged and, on the 38th day after the surgical intervention, he returned to the Orthopedics Clinic due to recurrence of pain on walking, an antalgic gait with auxiliary support, and local signs of inflammation at the level of the left knee joint.

Local examination of the left knee revealed a midline incision of approximately 15 cm centered on the patella, healed per primam, locally hyperemic skin, slightly increased local temperature, marked edema at the level of the left knee joint, areas of local dehiscence in the proximal, middle, and distal thirds of the surgical wound, discharging a moderate amount of serous-citrine fluid, hypotrophy of the thigh and calf musculature, pain-limited active and passive range of motion, flexion of 70 degrees, and an extension deficit of 10 degrees. On palpation, pain was absent in the perilesional tissues.

Blood samples were collected for complete blood count (CBC) analysis (Table 1). Laboratory findings included leukocytosis of 18.86 × 10^3^/L, with an increased number of neutrophils (16.43 × 10^3^/L), a procalcitonin of 0.5 µg/L and a CRP of 1.87 mg/dL.

Chemical and mechanical wound debridement was performed, and purulent discharge samples were collected for bacteriological examination, fungal identification, and antibiotic susceptibility testing, followed by a sterile local dressing. Based on the anamnestic data, the patient reported contact with the family dog, specifically, the dog accidentally licked the wound.

For microbiological diagnosis, the specimens were inoculated on the routinely used culture media: blood agar, Mannitol salt agar, CLED agar (Cystine–Lactose–Electrolyte-Deficient agar), Chocolate agar and Sabouraud dextrose agar.

Direct microscopy was also performed from the sample, revealing an increased number of neutrophils and erythrocytes and frequent Gram-variable cocci, as well as Gram-negative diplococci, raising the initial suspicion of a bacterial infection.

**Figure 1:**
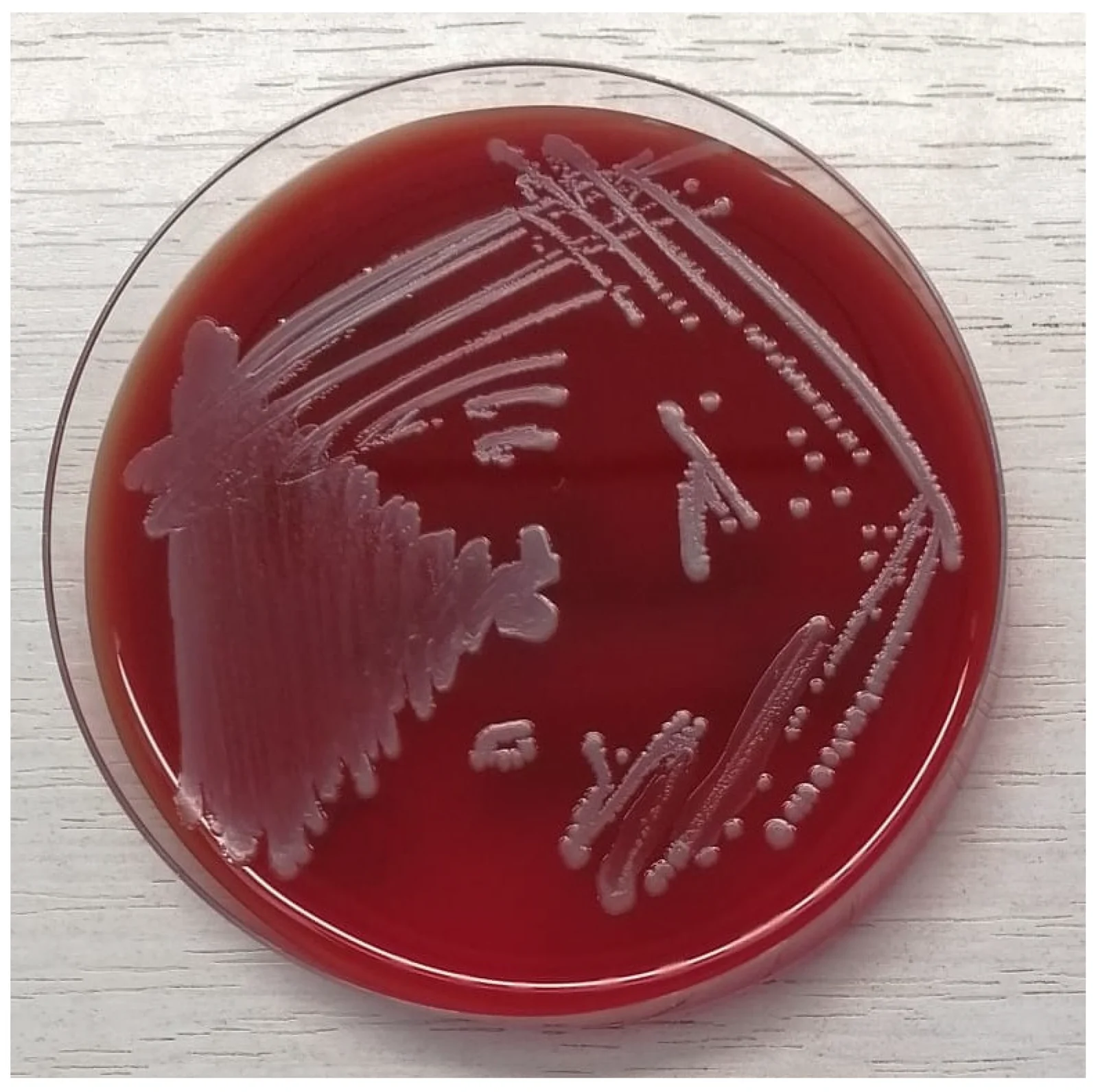
Direct culture on blood agar of the first purulent discharge sample collected from the patient. Smooth, grayish colonies grew in a rich, pure culture, which were later identified using MALDI-TOF MS as *Pasteurella canis*.

After 24 h of incubation, at 37 °C in an oxygen atmosphere (and a CO_2_-enriched atmosphere for Chocolate agar), an abundant culture of smooth, grayish colonies, catalase and oxidase positive was observed (Figure 1). Microscopy of the culture revealed Gram-negative coccobacilli.

Identification to the level of species was performed from the culture grown on blood agar using MALDI-TOF MS, based on the unique protein fingerprint of the bacterium, with *Pasteurella canis* as the result with an identification index over 2 (accurate identification to the level of species).

Antibiotic susceptibility testing (AST) was performed according to the EUCAST guidelines, on Mueller–Hinton agar with 5% defibrinated horse blood and 20 mg/L ß-NAD (MH-F), with a standardized 0.5 McFarland inoculum. The medium was incubated for 24 h at 37 °C in a 5% CO_2_-enriched atmosphere. The diameters were read from the front of the plate with the lid removed and with reflected light and then interpreted according to the latest EUCAST clinical breakpoint standard available.

**Figure 2:**
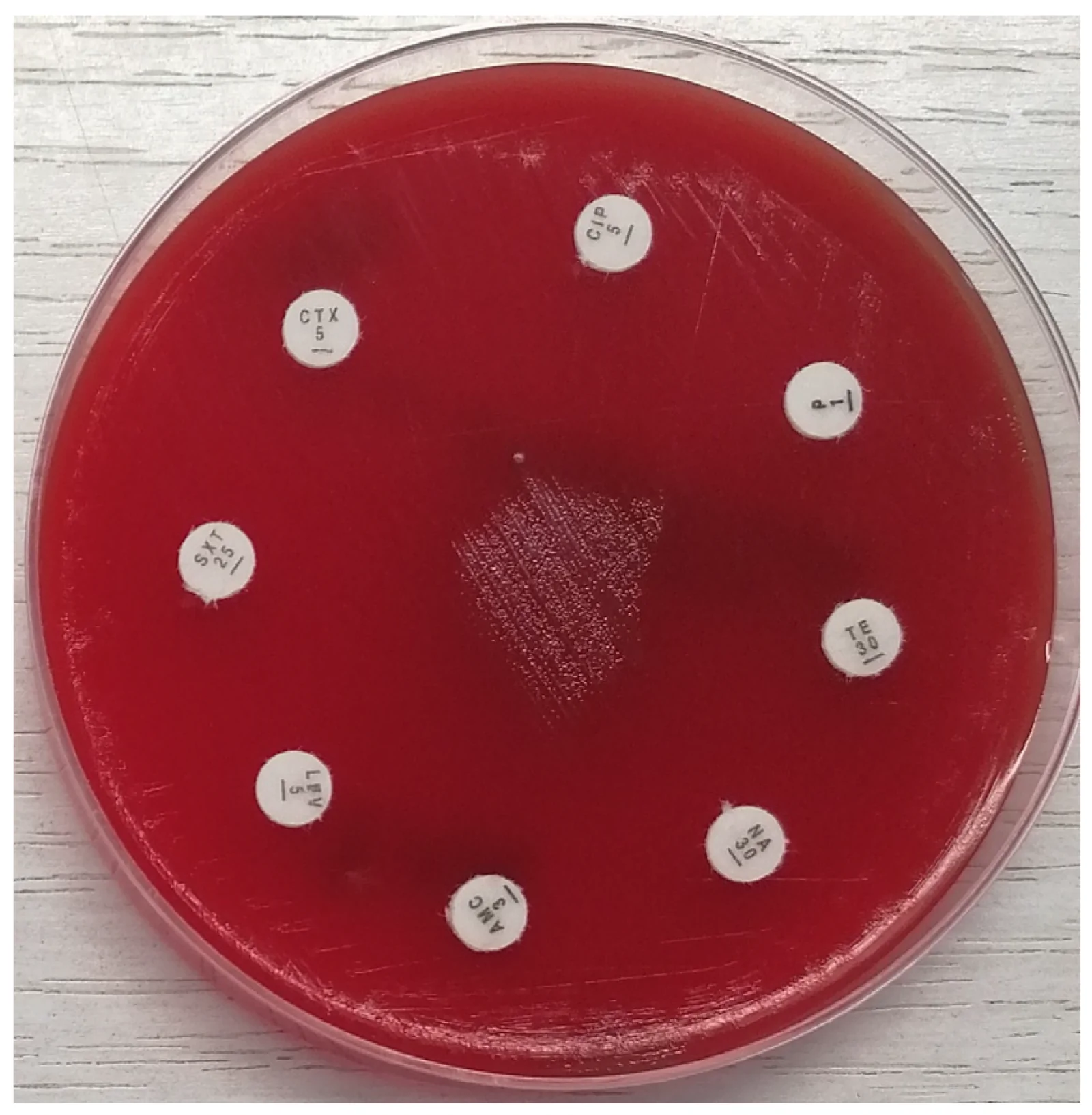
The results of the antibiotic susceptibility testing on Mueller–Hinton agar with 5% defibrinated horse blood and 20 mg/L ß-NAD after incubation for 24 h at 37 °C in a 5% CO_2_-enriched atmosphere. P—penicillin, TE—tetracycline, NA—nalidixic acid, AMC—amoxicillin and clavulanic acid, LEV—levofloxacin, SXT—trimethoprim-sulfamethoxazole, CTX—cefotaxime, CIP—ciprofloxacin. The clear area around the antibiotic disks represents the areas of inhibition, with all antibiotics tested being susceptible.

The strain was susceptible to all the tested antibiotics (Figure 2): penicillins (benzylpenicillin, amoxicillin and clavulanic acid), cephalosporins (cefotaxime), fluoroquinolones (ciprofloxacin, levofloxacin), tetracyclines and trimethoprim-sulfamethoxazole. The initial empiric treatment was adjusted according to the microbiological results.

At 48 h, another surgical and chemical debridement of the wound at the level of the joint was performed, and another purulent discharge sample was sent for microbiological testing. The sample analysis followed identical steps, being inoculated on the same culture media and incubated following the same protocol. The culture growth after 24 h was positive again for *Pasteurella canis* (identified from blood agar using MALDI-TOF MS with an identification index over 2), as well as some mucous colonies, which were lactose positive, that were later identified by automatic methods as *Klebsiella oxytoca,* but in a lower quantity. Antibiotic susceptibility testing was performed again according to the EUCAST guidelines, showing the same susceptibility to antibiotics for *Pasteurella canis* as before, as well as a strain of *Klebsiella oxytoca* susceptible to cephalosporins. Cefuroxime axetil was started orally at a dose of 250 mg twice daily (every 12 h) for 5 days, followed by 2 days of intravenous administration.

### 2.4. Third Admission (Infectious Diseases)

The patient was discharged at his own request, against medical advice, and was readmitted nine days later to an Infectious Diseases department with suspected septic arthritis of the left knee, superficial infection with wound dehiscence superinfected with *Pasteurella canis* and *Klebsiella oxytoca*.

Laboratory findings showed a persistently increased leukocytosis of 13.68 × 10^3^/L, with an increased number of neutrophils (8.56 × 10^3^/L), a procalcitonin of 0.5 µg/L, a CRP of 5.31 mg/dL and a fibrinogen of 742 mg/dL (Table 1).

A third pus sample collected from the wound discharge isolated, in addition to *Pasteurella canis* and *Klebsiella oxytoca*, *Pseudomonas aeruginosa*, susceptible to ceftazidime, and the antibacterial treatment was changed accordingly, to ceftazidime (1 g) and gentamicin (80 mg/2 mL), administered intravenously three times daily (every 8 h). The clinical course of the disease under treatment was favorable, and imaging investigations ruled out involvement of the prosthetic joint.

**Figure 3:**
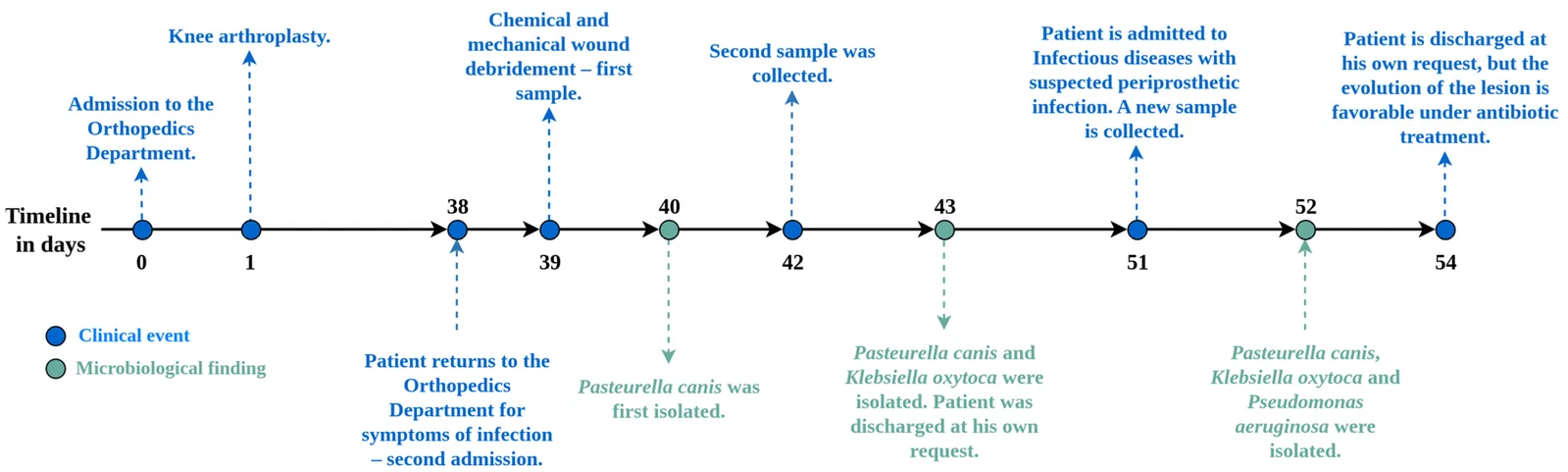
Timeline for an immunocompetent patient who developed periprosthetic joint infection with *Pasteurella canis*, later associated with *Klebsiella oxytoca* and *Pseudomonas aeruginosa*.

An overview of the patient’s timeline during his hospital admissions can be seen in Figure 3.

## 3. Discussion

We report a case of periprosthetic joint infection with *Pasteurella canis* in a patient with cardiovascular comorbidities, who underwent a total knee replacement 38 days prior to developing symptoms of post-surgical infection. The patient admitted to having a dog who accidentally licked the wound before he was admitted to the hospital for a second time with symptoms of infection. After surgical and chemical debridement of the lesion, samples were collected and sent for microbiological diagnosis, revealing an abundant culture of *Pasteurella canis* as the etiological agent of the infection. During his future admissions to the Orthopedics and Infectious Diseases departments, two more samples were collected, in which *Pasteurella canis* was isolated, along with *Klebsiella oxytoca* (second sample), and *Klebsiella oxytoca* and *Pseudomonas aeruginosa* (third sample). Patient evolution was favorable under targeted antibiotic therapy according to AST.

Approximately 15–20% of dog bites and more than 50% of cat bites contain a mixture of aerobic and anaerobic bacteria that are commonly found in the normal flora of their oral cavity [19]. Talan et al. [4] published a comprehensive study on the aerobic and anaerobic bacteria found in dog bites, with *Pasteurella* spp. being present in 50% of the wounds, followed by *Streptococcus* spp. (46%), *Staphylococcus* spp. (46%), *Fusobacterium* spp. (32%) and *Bacteroides* spp. (30%). Empiric antibiotic therapy should cover those pathogens when dog or cat bites (or contact of the wound with animal saliva) are suspected. Therefore, beta-lactams or beta-lactamase inhibitors, along with second generation cephalosporins or combination therapies with penicillin and first generation cephalosporins or clindamycin or a fluoroquinolone are recommended as first-line therapy [20]. The first line of treatment in infections with *Pasteurella canis* is with penicillins, or, alternatively, with fluoroquinolones, doxycycline, or trimethoprim-sulfamethoxazole [1].

*Pasteurella* spp. severe forms of infection can occur in healthy individuals, but immunocompromised individuals, children or the elderly have a higher risk. Prosthetic joints increase the risk of infection in the cases of septic arthritis, specifically in patients with diabetes or immunosuppression. Patients with bacteriemia due to *Pasteurella* spp. have an increased mortality, but in people with periprosthetic joint infections, the prognostic is good with full recovery [1].

The most recent case of infections with *Pasteurella* spp. described in the literature is also one of the most severe ones. Pefferkorn et al. (2026) [21] described the case of a 25-year-old man who developed otitis a few days after his dog licked his ear, and died at home about 20 days later after a febrile illness with headaches. Autopsy showed purulent meningitis and cultures from the ear and a subdural empyema grew *Pasteurella canis* together with *Streptococcus anginosus* group. It is the first reported fatal case of central nervous system infection with this pathogen in an adult. The case highlights two important points: that a microorganism that is usually considered a harmless animal commensal can occasionally cause life-threatening infections in humans and that close contact between humans and pets, including seemingly harmless behaviors such as licking, can become a route of transmission for zoonotic pathogens, particularly when local tissue damage or infection is present, similar to the current case.

Microbiological diagnosis of *Pasteurella canis* relies on cultivating the microorganism, microscopic examinations and biochemical or automated identification systems (MALDI-TOF MS, Vitek 2C). Diagnosing this infection can be challenging, but extremely important, as it can help guide targeted antimicrobial therapy, especially in immunosuppressed patients with multiple comorbidities. Raising the suspicion of a diagnosis with unusual microorganisms and requesting the appropriate laboratory tests while performing the surgical debridement of the wound is crucial to be able to reveal the correct etiology of these infections.

Specimen collection is according to the symptoms (e.g., pus, joint fluid, blood cultures, sputum, etc.). Direct microscopy from the specimen can raise the suspicion (Gram-negative coccobacilli). *Pasteurella* spp. grow well on 5% sheep blood agar and Chocolate agar, and not so well on MacConkey agar. Incubation is done in aerobic conditions, at 37 °C for 18 ± 2 h. Colonies have a characteristic aspect: smooth, grayish-white, catalase and oxidase positive [20].

A recent case series and comprehensive literature review published by Chisala et al. (2026) [17] present three cases of prosthetic joint infections with *Pasteurella* spp. From the three cases presented by the authors, two were infections with *Pasteurella multocida* and only one was *Pasteurella canis*, isolated over a timespan of ten years (28 February 2013 and 28 February 2023). The authors also conducted a comprehensive literature search and found a total of 57 cases of prosthetic joint infections with *Pasteurella* spp. between 1975 and 2024, with 56 of those cases being *Pasteurella multocida* and one case *Pasteurella canis.* A comparative overview of the present case, compared to the ones that were found in the literature, can be seen in Table 2.

The present case report has several limitations. The exact cause of the infection was never confirmed, as no samples were taken from the oral cavity of the patient’s dog to describe its oral flora and check if *Pasteurella canis* was present. Furthermore, since the strain was never isolated from the dog, the strains from the wound and the ones from the oral cavity of the animal could never be compared by molecular methods to fully prove the causative agent of the infection. The patient mentioned that he owned a dog, who accidentally licked the wound, but did not specifically state how many times the dog came in contact with the wound or what were the exact conditions that led to the wound getting infected. However, considering that *Pasteurella* spp. are zoonotic pathogens, the dog being the source of the infection remains the most plausible theory.

## 4. Conclusions

Zoonotic diseases, such as pasteurellosis, should be considered especially in people who have wounds that come in close contact with their dog or cat, such as bites, licks or scratches, and develop symptoms of infection. As the range of infections caused by *Pasteurella* spp. is very complex and can become life-threatening in immunosuppressed or elderly patients, appropriate management is mandatory and requires interdisciplinary collaboration for appropriate diagnosis. Periprosthetic infections, like the one presented in the current case, are frequently polymicrobial, requiring microbiological diagnosis and targeted antimicrobial therapy for complete recovery.

## Figures and Tables

**Table 1:** Laboratory findings during the hospital admissions of the patient. The increased values are marked in bold.

Laboratory Test	Normal Range	First Admission	Second Admission	Third Admission	Follow Up
**RBC (10^6^/µL)**	4.0–5.5	4.72	4.01	4.18	3.22
**Hematocrit (%)**	40–54	45	36.3	36.8	35.8
**Hemoglobin (g/dL)**	12–16	15.9	12.9	13.2	13
**Lymphocytes # (10^3^/µL)**	0.8–4	3.13	1.47	3.46	3.40
**Lymphocytes (%)**	20–40	29.6	7.8	25.3	31
**Monocytes # (10^3^/µL)**	0.12–1.2	0.70	0.81	0.90	0.8
**Monocytes (%)**	1–10	6.6	4.3	6.6	6.5
**Neutrophils # (10^3^/µL)**	1.5–7	6.48	**16.43**	**8.56**	5.8
**Neutrophils (%)**	50–70	61.4	**87.1**	64.8	52
**Leucocyte # (10^3^/µL)**	4.0–10	9.3	**18.86**	**13.68**	7.6
**Platelets # (10^3^/µL)**	150–400	293	323	**676**	300
**CRP (mg/dL)**	0.5	0.23	**1.87**	**5.31**	0.45
**Fibrinogen (mg/dL)**	170–420	200	230	**742**	390
**Procalcitonin (µg/L)**	<0.5	<0.5	<0.5	<0.5	<0.5

#—absolute value, RBC—red blood cells.

**Table 2:** Overview of the current case compared to other cases of periprosthetic joint infections with *Pasteurella canis* reported in the literature.

Study	Gender	Age	Comorbidities	Animal Contact	Location of Animal Contact	Time from Surgery to Infection	Affected Joint	Symptoms	Management	**Antibiotic Used and Duration**	**Outcome**
Chisala et al. (2026) [17]	Male	47	Gout, arthritis, diverticular abscess	Dog	Dog licking the surgical wound	1 month	Right knee	Pain, swelling, erythema, wound discharge	Surgical knee aspiration, debridement, antibiotics	IV cefuroxime and teicoplanin (penicillin allergy)—2 weeks; 4 weeks of oral doxycycline 200 mg and flucloxacillin 500 mg	cured
Mondon et al. (2005) [18]	Woman	77	Ankylosing spondylitis	Dog	Dog bite on the left hand	2 and a half years after second surgery	Right knee	Cellulitis of the right leg	Surgical irrigation and debridement of the prosthesis, antibiotics	Intravenous penicillin G—8 days, followed by amoxicillin—10 days, after culture—levofloxacin and amoxicillin	cured
Current study	Male	62	Hypertension	Dog	Dog licking the surgical wound	38 days	Left knee	Pain, swelling, erythema, wound discharge	Surgical and chemical debridement of the wound	Cefuroxime, followed by Ceftazidime (polymicrobial wound)	cured

## Data Availability

All available data regarding the case is presented in the text of the manuscript.

## Abbreviations

The following abbreviations are used in this manuscript:

AST	Antibiotic susceptibility testing
CBC	Complete blood count
CLED	Cystine–Lactose–Electrolyte-Deficient agar
CRP	C-reactive protein
EUCAST	European Committee on Antimicrobial Susceptibility Testing
MALDI-TOF MS	Matrix-Assisted Laser Desorption/Ionization Time-of-Flight Mass Spectrometry
RBC	Red blood cells
